# Real-Time Definition of Non-Randomness in the Distribution of Genomic Events

**DOI:** 10.1371/journal.pone.0000570

**Published:** 2007-06-27

**Authors:** Ulrich Abel, Annette Deichmann, Cynthia Bartholomae, Kerstin Schwarzwaelder, Hanno Glimm, Steven Howe, Adrian Thrasher, Alexandrine Garrigue, Salima Hacein-Bey-Abina, Marina Cavazzana-Calvo, Alain Fischer, Dirk Jaeger, Christof von Kalle, Manfred Schmidt

**Affiliations:** 1 Department of Translational Oncology, National Center for Tumor Diseases, Heidelberg, Germany; 2 Department of Medical Biostatistics, Tumor Center Heidelberg-Mannheim, Heidelberg, Germany; 3 Molecular Immunology Unit, Institute of Child Health, University College London, London, United Kingdom; 4 Department of Clinical Immunology, Great Ormond Street Hospital NHS Trust, London, United Kingdom; 5 INSERM Unit 768, Hôpital Necker and Faculté de Médecine Université René Descartes Paris V., Paris, France; 6 Département de Biothérapies, Hôpital Necker, Paris, France; 7 Unité d'Immunologie et d'Hématologie Pédiatriques, Hôpital Necker, Paris, France; 8 Division of Experimental Hematology, Cincinnati Childrens Research Foundation, Cincinnati, Ohio, United States of America; National Cancer Institute at Frederick, United States of America

## Abstract

Features such as mutations or structural characteristics can be non-randomly or non-uniformly distributed within a genome. So far, computer simulations were required for statistical inferences on the distribution of sequence motifs. Here, we show that these analyses are possible using an analytical, mathematical approach. For the assessment of non-randomness, our calculations only require information including genome size, number of (sampled) sequence motifs and distance parameters. We have developed computer programs evaluating our analytical formulas for the real-time determination of expected values and *p*-values. This approach permits a flexible cluster definition that can be applied to most effectively identify non-random or non-uniform sequence motif distribution. As an example, we show the effectivity and reliability of our mathematical approach in clinical retroviral vector integration site distribution.

## Introduction

With the sequences of complete genomes available [1]–[4], and accelerating technologies for high-throughput sequencing [5] genome wide sequence analyses of individual samples will soon become reality. Comparative analyses of sequence composition and sequence motif distribution have become central parts of genome and transcriptome research, providing new insights on evolution, physiology and medical diagnosis [6]–[15]. Our understanding of integrating viruses and related vectors in gene therapy trials is an interesting example of such approaches. Since the completion of the human and murine genome sequencing projects the location of the vector in the cellular genome can be defined precisely, allowing the determination of possible vector integration induced effects on the surrounding genomic DNA regions at the molecular level. Integration site analyses have gained increasing interest with the dramatic development of a retroviral vector-induced lymphoproliferative disease in 3 patients cured of X-linked severe combined immunodeficiency (X-SCID) that was triggered by insertional activation of the proto-oncogene *LMO2* [16], [17]. Meanwhile, insertion induced side effects have been identified ranging from immortalization [18] to clonal dominance [19]–[22] and even oncogenesis [23]–[25] in a variety of gene therapy studies. These studies have in common that a clustering of integration sites (IS) in certain genomic loci was detectable, and likely provided a selective advantage for the affected cell clone.

The clustering of integrations, termed common integration sites (CIS), as an indicator for clone selection has already been used in concerted retrovirus insertional mutagenesis studies that aimed to identify new cancer genes by determining the gene configuration near frequently affected integration site loci [26]–[28]. For CIS determination, computer simulations were performed to assess non-randomness of IS distribution in tumors [28]. To validate the correctness of our mathematical approach defining non-randomness and non-uniform sequence motif distribution, we analyzed the IS distribution and presence of CIS in 2 successful clinical SCID-X1 studies [29,30, unpublished data]. We considered 2, 3 or 4 insertions as CIS of 2^nd^, of 3^rd^ or 4^th^ order if they fell within a 30 kb, 50 kb or 100 kb window of genomic sequence from each other, respectively. Simultaneously, we performed computer simulations written in open source ‘R’-language (http://cran.r-project.org) for which a window of size *d_n_* (*d_n_*  =  the maximum distance defining a CIS of order *n*) was shifted through the ordered sequence of the IS. For each window W(*j*) = [IS(*j*),IS(*j*)+*d_n_*] it was then counted how many CIS of order *n* including IS(*j*) as first element were contained in W(*j*). We show that our mathematical approach for defining biased IS distribution is comparable to the output of computational simulations. It may have advantages in performance of large quantities of individual analyses. Even if the null hypothesis of random uniform allocation is not adequate, as it is known from retroviral vector integration [31], our calculations can address segments of the genome located between sites of predilection for virus integration and can be extended to address non-uniform sequence motif distributions.

## Results and Discussion

### Part 1: Random uniform allocation of IS

For the purpose of this discussion, the unit of observation (location and distance) is kilobasepair (kb). We assume that a number *n*_is_ of IS is randomly allocated (with a uniform distribution) to the locations of a genome consisting of *g* kb. A CIS of order *n* is an *n*-tuple of IS such that the maximum distance between the lowest and highest position is no greater than a fixed bound.

Further terminology

*d_n_*: defining “size” or distance of a CIS of order *n*, i.e. maximum permissible distance between any two members of a CIS of order *n*
*P_n_*: probability that a given (sub)set of *n* IS that are randomly allocated form a CIS of order *n*
*P*(*m,d*): probability that a given subset of *m* randomly allocated IS has a span ( =  maximum distance between any two elements) of exactly *d*
*E_n_*: expected value of the number of CIS of order *n*

We start with the elementary observation that *E_n_* equals *P_n_* times the number of subsets of IS consisting of *n* elements:
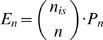
(1)Clearly,
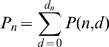
(2)It remains to determine *P*(*n,d*). First note that *P*(1,*d*) = 0 for *d*>0. Furthermore, for all *m*≥1:
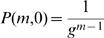
(3)


A recursive formula for *P*(*m,d*), *d*>0, can be derived by breaking down the potential CIS of order m into subsets of *m*–1 elements having a span of *d*'≤*d*, to which an *m*-th IS is added such that the maximum span is exactly *d*:
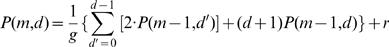
(4)where *r* is a negligible correction term that arises because the uncorrected recursion formula is strictly valid only for subsets of IS that have a distance ≥*d* from the telomeres.

By mounting the recursive ladder (*m* = 1,...,*n*), these formulas successively yield *P*(*n,d*), *P_n_*, and *E_n_*. In particular, one easily obtains (*d*>0):
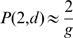


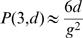


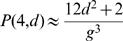
Plugging this into equations (2) and (1) yields for the expected value *E_n_*:
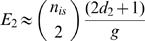


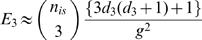


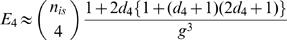
As shown in **Table 1**, our mathematical approximation corresponds extremely well to the mean values found in 50000 simulation runs.

**Table 1:** Mean values for random CIS formation (1000 IS) determined either with computer simulations or mathematically.

Order of CIS	Mean Value Mathematical Formula	Mean Value Computer Simulations
2^nd^	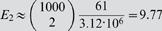	9.75
3^rd^	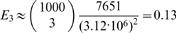	0.13
4^th^	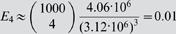	0.01

Simulations were performed with 50000 runs each. g, haploid size of the human genome: 3.12 x 10^6 ^kb; *d*_n_, genomic window size [kb] for CIS of n^th^ order: *d*_2_ = 30, *d*_3_ = 50, and *d*_4_ = 100; *n*_is_, number of (assumed) sampled integration sites: 1000.

Statistical inferences, such as the calculation of *p*-values, can be based on the observation that, under the null hypothesis (*H*_0_) of random uniform allocation of the IS, the number of CIS of order *n* is (approximately) Poisson distributed with parameter *λ* = *E*_n_. Thus, if the random variable *X* denotes the number of CIS of order *n*, and *X* = *k* is observed in a trial, then the *p*-value *P*(X≥k) of this observation calculated under *H*_0_, i.e. from the Poisson distribution *P*_o_(*E_n_*), is given by
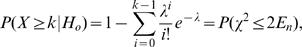
where the random variable *χ^2^* has a chi-square distribution with 2 k degrees of freedom [32], [33].

The Poisson approximation to the true random distribution of CIS is exceedingly close. In fact, if the number of simulation runs is sufficiently high, the simulated distribution is virtually undistinguishable from *P*_o_(*E_n_*). In particular, both the expected values and the *p-*values derived from *P*_o_(*E_n_*) are nearly identical to those obtained in computer simulations. The latter point is apparent from **Table 2**, where for a final proof of principle of our mathematical calculations, results of the analysis of our integration data set retrieved from two clinical SCID-X1 therapy trials [unpublished data] are given.

The *p*-value can be calculated by means of either of the following commands (‘R’ code): 1–ppois(lambda = *E_n_*, *q* = *k*–1) or pchisq(d*f* = 2*k*, *q* = 2*E_n_*). Using the data of **Table 2** (first line) 1–ppois(lambda = 0.19, *q* = 2) or pchisq(df = 6, *q* = 0.38). In both instances, the result is 0.00099. Alternatively, the table of the chisquare distribution with 6 degrees of freedom can be used to look up the probability *P*(*X*≤0.38). One should note that, for low *E_n_*, the *p*-value of a single observed CIS is virtually identical to *E_n_*. This implies that, for *n*>5, no *p*-values need to be calculated (and hence no formulas are required for *E_n_*, *n*>5), because even with an extremely liberal definition of the CIS (*d*_5_ = 500) and a fairly high number of IS (*n*_is_ = 1000) a single CIS of order 5 will be statistically significant (*p* = 0.027).

**Table 2:** Comparative analysis of mean values and *p-*values obtained computationally (‘Simulation’) or mathematically (‘Formula’).

CIS	IS	MV Simulation	MV Formula	*p*-Value Simulation	*p*-Value Formula
3	140	0.188	0.190	0.0009	0.001
1	134	0.175	0.174	0.16	0.16
4	102	0.100	0.101	0	3.9×10^−6^
15	304	0.899	0.900	0	6.8×10^−14^
102	572	3.200	3.193	0	<10^−16^

The results refer to the presence of CIS detected in 2 clinical X-SCID gene therapy studies [unpublished data]. Simulations were performed with 50000 runs on the haploid size of the human genome (3.12×10^6^ kb). *P-*values estimated from simulations equal the proportion per 50000 runs in which the number of CIS was at least as high as the number observed in the trials. The genomic window size chosen for CIS of 2^nd^ order was 30kb. CIS, number of identified CIS of 2^nd^ order in patient and control samples pre- and post-transplant; IS, number of all unique identified integration sites in patient and control samples pre- and post-transplant; MV, mean value.

### Part 2: Non-uniform allocation of IS

Defining non-randomness in the clustering of genomic events often requires additional precautions as sequence structures of interest may already have known specific distribution biases. In the case of our clinical example (unpublished data), it is known that retroviral vectors based on the murine leukaemia virus (MLV) tend to integrate into gene coding regions preferentially near the transcriptional start site (TSS) [34]-[36]. It is also proposed that additional factors, indeed mostly unknown, may influence the accessibility of vectors to certain genomic DNA regions [37]. Thus, the null hypothesis of random uniform allocation of MLV IS distribution may not be adequate according to the current ‘state of the art’, as has recently been argued [31]. In line with this study, we portioned the genome into 2 adequate areas that differ in the likelihood of getting targeted by vectors.

Further terminology

*n*_TSS_: number of TSS
T5: an interval of +/-5kb around a TSS
GT5: union of all T5
*n*_is,Mix_, *n*_is,Comp_: number of IS occuring in GT5 and in the complement of GT5, respectively
*n*_cis,GT5_, *n*_cis,Mix_, *n*_cis,Comp_: number of CIS occurring in GT5, both in GT5 and in the complement of GT5 and in the complement of GT5 only, respectively

Clearly, the expected value *E*_n_ of the number CIS of order *n* is given by the following sum:
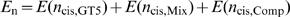
(5)In the following it will be shown how to calculate the terms on the right side of (5). We start with the expected value of *n*_cis,GT5 _fore what we assume that vector integration into any T5 occurs with the same probability. Then
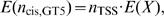
(6)where *X* is the number of CIS (among those occurring in GT5) that occur in a fixed T5. Observing that *i* IS in a fixed T5 yield 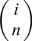 CIS of order *n* in this T5 one easily obtains the expected value of *X*

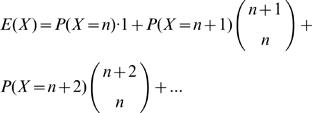
(7)Since X is binomially distributed as ∼ *B*(*n*_is_,GT5,1/*n*_TSS_),
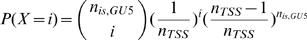
(8)Merging equations (6)–(8) yields the desired formula for *E*(*n*_cis,GT5_):
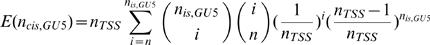
(9)


If *n*_is,GT5_ is small compared to *n*_TSS_ (undoubtedly, this is mostly the case), terms of higher order can be neglected so that, because (*n*_TSS_–1)/*n*_TSS_≈1, formula (9) simplifies to
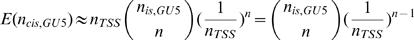
(10)


Notice that formulas (6)–(10) do not depend on the spatial distribution of the IS within the T5. (It is unnecessary to account for the closeness of IS within T5 because *any* pair – or triple, quadruple etc., for that matter – of IS within a T5 yields a CIS.)

Clearly, the expected value of *n*_cis,Mix_ *E*(*n*_cis,Mix_) is not independent of the distance between the IS and the TSS. Thus, inevitably, assumptions regarding the spatial distribution for the IS will influence its value. In the sequel, a formula for *E*(*n*_cis,Mix_) shall be derived for the case *n* = 2. As before, CIS of order 2 are defined by a maximum distance *d*_2_ of 30kb between the IS.

If the TSS are indistiguishable with respect to the probability distribution of the integrations, then
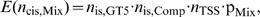
(11)where *p*_Mix_ denotes the probability that an arbitrary pair of IS (with one element in GT5 and one element in the complement of GT5) forms a CIS of order 2 around a fixed TSS.

We will assume that the distributions of IS within a T5 and within +/-35 kb around a TSS are symmetric. Then, again using kb as unit of distance,
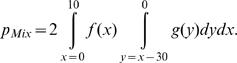
(12)


In formula (12) the points *x* = 0 and *y* = 0 correspond to the TSS-5; *f*(*x*) designates the probability density function of vector integrations in T5; and *g*(*y*) designates the corresponding density function in [TSS-35, TSS-5].

Formula (12) shall be evaluated for two special cases:

**Case 1**: Vector integrations are uniformly distributed in GT5 and in the complement of GT5, respectively. I.e.,
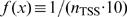


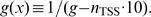
Solving the integrals in formula (12) we have
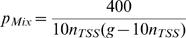
(13)


**Case 2**: As above, vector integrations in the complement of GT5 are assumed to be uniformly distributed. However, a triangular distribution is assumed for *f*(*x*). The corresponding formula is easily calculated:
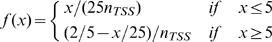
By plugging this into (12) we get
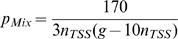
(14)


It may be surprising that a triangular distribution in T5 results in a higher expected value for *n*_cis,Mix_ than a uniform distribution. However, this becomes more plausible if one notes that a higher value is also obtained if the IS are concentrated in an extreme manner within the T5, viz. in a one-point distribution with total mass in the TSS. In this special case (which is particularly easy to evaluate), *p*_Mix_  =  50/(*n*_TSS_(*g–n*_TSS_)).

If, with respect to the formation of CIS, the complement of GT5 could be regarded as a continuum, the expected value of *n*_cis,Comp_ would be given by the formulas developed in **Part 1** of this contribution. In the case of retroviral (MLV) vectors, however, the complement of GT5 has rather to be viewed as a partitioned set consisting of approximately TSS disjoint intervals. It follows that that the residual term on the right-hand side of equation (4) (**Part 1**) may no longer be negligible. Note however, the assumption of a continuum clearly tends to lead to an overestimation of the number of CIS, because the boundaries of the components reduce the number of CIS occurring in their neighborhood. It follows that the formulas derived in **Part 1** form an upper bound for *E*(*n*_cis,Comp_). In particular, the true *p*-values are less or equal to the values calculated by means of the formulas derived in **Part 1**. Therefore, any positive statements regarding statistical significance remain valid. Moreover, the overestimation is probably fairly small given that the sections of GT5 located between the TSS are mostly rather wide compared to the length defining a CIS.

Indeed, the null hypothesis of non-uniform allocation for IS distribution does not substantially change the results we have obtained based on the hypothesis of a random uniform allocation for CIS formation in our clinical samples **(** **Table 2** **)**, as is shown in **Table 3**.

**Table 3:** Formulas based statistical analysis of the results on CIS formation in clinical samples derived from 2 clinical X-SCID gene therapy studies [unpublished data].

CIS	IS	MV Uniform^*^	MV Triangular^§^	*p*-Value Uniform*	*p*-Value Triangular^§^
3	140	0.191	0.212	0.001	0.0014
1	134	0.175	0.195	0.161	0.177
4	102	0.101	0.124	4.0 x 10^−6^	6.1 × 10^−6^
15	304	0.905	1.006	7.4 × 10^−14^	3.3 × 10^−13^
102	572	3.212	3.568	<10^−16^	<10^−16^

Calculations were performed on the haploid size of the human genome (3.12 × 10^6^ kb) and on the basis of an IS skewing (25% of all IS) to the +/− 5 kb TSS region, for which an (*) uniform or a (^§^) triangular IS distribution, respectively, was assumed. 75% of IS were assumed to be uniformly distributed over the remaining human genome. The genomic window size chosen for CIS of 2^nd^ order was 30 kb. CIS, number of identified CIS of 2^nd^ order in patient and control samples pre- and post-transplant; IS, number of all unique identified integration sites in patient and control samples pre- and post-transplant; MV, mean value.

Our mathematical formulas allow a reliable, straightforward calculation of non-randomness in CIS and other genomic event distributions under the null hypothesis of uniform and non-uniform allocation. Using formula based workspaces (available on request), expected values and *p*-values can be calculated with ease in real-time. They may be preferable to computer simulations when (routine) high-speed processing of large quantities of analyses is needed. Our approach enables a closely problem-oriented, highly exact evaluation of non-randomness that is useful for assessing IS distribution in clinical trials and for assessing the distribution of any sequence motif of interest in a natural or artificial genome.
